# EVALUATING THE ACCEPTABILITY AND PRELIMINARY EFFECTS OF EMPOWER@HOME FOR GERIATRIC DEPRESSION

**DOI:** 10.1093/geroni/igad104.2147

**Published:** 2023-12-21

**Authors:** Jay Kayser, Skyla Turner, Chuxuan Zheng, Samson Ash, Xiaoling Xiang

**Affiliations:** University of Michigan, Ann Arbor, Michigan, United States; University of Michigan, Ann Arbor, Michigan, United States; University of Michigan, Ann Arbor, Michigan, United States; University of Michigan, Ann Arbor, Michigan, United States; University of Michigan, Ann Arbor, Michigan, United States

## Abstract

Internet-based cognitive behavioral therapy is a cost-effective strategy for meeting older adults’ increased mental health needs, but few existing programs are designed for older adults. Our team developed Empower@Home using user-centered design and community-engaged approaches. This poster presents results from year 1 of our ongoing uncontrolled trial (NCT05384704). Participants were recruited from a research volunteer registry, and community agency referrals. 104 adults 50+ (M=63) consented and started the intervention in 2022, of which 96 completed the post-test and a 10-week follow-up (93% retention rate). Items from the Modified Treatment Evaluation Inventory showed high satisfaction and acceptability. For example, 95.5% agreed that the program was “an acceptable way of dealing with depressed moods,” and 96.8% agreed that they “would recommend this program to others who experience depressed moods.” Adherence was high, with an average of 8.5 sessions (out of 9 total) completed (n=103). Among those who completed the post-test (n=96), all but one finished the entire program. Intention to treat analysis showed a significant within-group moderate reduction in depressive symptoms as measured by the PHQ-9 from pre to post-test (cohen’s d=.75). This effect size was large among those with a pre-test PHQ-9 score of ≥10 (cohen’s d=1.31). The treatment effect was largely maintained at the 10-week follow-up. Significant improvements in post-test were also observed for secondary clinical outcomes, including anxiety (GAD-7), social isolation (DSSI), loneliness (PROMIS), Anger (PROMIS), and sleep quality. Empower@Home is an acceptable and potentially effective program for treating depression in older adults.

